# Structural and Chemical Properties of Geopolymer Gels Incorporated with Neodymium and Samarium

**DOI:** 10.3390/gels7040195

**Published:** 2021-11-03

**Authors:** Snežana S. S. Nenadović, Ljiljana M. Kljajević, Marija M. Ivanović, Miljana M. Mirković, Nadežda Radmilović, Lazar Z. Rakočević, Miloš T. Nenadović

**Affiliations:** 1 Department of Materials, Vinča Institute of Nuclear Sciences, National Institute of the Republic of Serbia, University of Belgrade, Mike Petrović Alasa 12-14, Vinča, 11000 Belgrade, Serbia; msneza@vin.bg.ac.rs (S.S.S.N.); ljiljana@vin.bg.ac.rs (L.M.K.); marija@vin.bg.ac.rs (M.M.I.); miljanam@vin.bg.ac.rs (M.M.M.); 2 Department of Atomics Physics, Vinča Institute of Nuclear Sciences, National Institute of the Republic of Serbia, Mike Petrović Alasa 12-14, Vinča, 11000 Belgrade, Serbia; nadezdas@vin.bg.ac.rs (N.R.); lazarrak@gmail.com (L.Z.R.)

**Keywords:** geopolymer gel, rare earth, Nd_2_O_3_, Sm_2_O_3_, thermodynamic parameters, DRIFT, XRD, XPS, TEM

## Abstract

The present work was focused on doping of 1% and 5% both of Nd_2_O_3_ and Sm_2_O_3_ in geopolymer gels. One of the main goals was to determine the influence of the behavior of Nd and Sm as dopants and structural nanoparticles changes of the final geopolymer formed. It is shown that the disorder formed by alkali activation of metakaolin can accommodate the rare earth cations Nd^3+^ and Sm^3+^ into their aluminosilicate framework structure. The main geopolymerization product identified in gels is Al-rich (Na)-AS-H gel comprising Al and Si in tetrahedral coordination. Na^+^ ions were balancing the negative charge resulting from Al^3+^ in tetrahedral coordination. The changes in the structures of the final product (geopolymer/Nd_2_O_3_; Sm_2_O_3_), has been characterized using X-ray diffraction (XRD), Fourier transform infrared spectroscopy (FTIR), X-ray photoelectron spectroscopy (XPS) and transmission electron microscopy (TEM) analysis with energy dispersive spectrometry (EDS). Nucleation at the seed surfaces leads to the formation of phase-separated gels from rare earth phase early in the reaction process. It is confirmed that Nd and Sm have been shown to form unstable hydroxides Nd(OH)_3_ and Sm(OH)_3_ that are in equilibrium with the corresponding oxides.

## 1. Introduction

A geopolymers are well-known alkali aluminosilicate cement materials that can have superior mechanical, chemical and thermal properties when compared to other Portland-based cements, showing significantly lower levels of CO_2_ production [1]. Geopolymers form a structure closed cage-like cavity together with combination of ring molecules that can separate metal ions or other toxic substances and catch them inside the cavity. Despite that, some metal cations can even participate in the geopolymerization reaction [2,3]. Research to date has shown a lot of results in field of toxic heavy metals treatment by immobilization into alkali activated structure [4,5,6,7,8,9,10,11,12].

Rare earth elements (REEs) have become increasingly important metals used in modern technology and potential applications. However, their increasing use in the industrial sector, medicine and agriculture over the last few decades has provided them with the title of “new pollutants” [13]. In the past few years, modification with some rare earth (RE) metals has shown to be an efficient method of improving the photocatalytic properties of TiO_2_ and broadening its absorption band within the solar spectrum [14,15]. Doped titania gels are prepared where presence of neodymium stabilizes the amorphous phase of the sample up to higher temperatures (400 °C) as compared to the other one containing samarium [16]. It is also confirmed that Sm^3+^ ions increase the surface area but also enhance the photocatalytic activity under UV or solar light irradiation on Sm-doped TiO_2_ [16,17,18,19]. With reference to organic dyes, it was established that the presence of Sm is highly effective against Methyl orange [20]. Samarium has been studied extensively as a dopant in a wide number of host materials because of its chemical properties and the considerable modification on the properties of the host that are measured when even small concentrations of samarium are present. Physical properties of glass samples can be improved by the addition of different modifiers between the other rare earth oxides (Gd_2_O_3_, Sm_2_O_3_, etc.) [21]. Rare earth oxide modifiers showed a significant improvement over glass systems. Gafara et al., 2020 investigated the structure and properties of Nd-modified Bi-Sr-borate glass, i.e., the effect of Nd_2_O_3_ addition on Bi-Sr-borate glasses for their possible applications in many aspects [22].

Rare earth oxides have been broadly utilized in different research areas due to their unique and interesting properties. Samarium oxide (Sm_2_O_3_) is one of the promising candidate materials among rare earth oxides because of some outstanding properties such as large conduction offset with Si, high thermal stability, small frequency dispersion and low trapping rates [23,24]. Sm_2_O_3_ is also predicted to be thermodynamically stable on Si substrates [25]. Neodymium oxide (Nd_2_O_3_) was selected for electronic applications not only because of its high κ but also good step coverage and good dielectric strengths [26]. In accordance with the above, samarium and neodymium oxides are important rare earth materials because of their suitability for optical, ceramic, solar cells, nanoelectronics, semiconductors, sensors and catalytic applications [27]. However, the risks of REE pollution due to mining and processing as well as from the improper disposal of materials containing these compounds could potentially lead to elevated levels within the environment. In addition, the processing of REE-rich monazite rocks for the production of phosphate fertilizers and the subsequent applications of these fertilizers could further elevate REE soil concentrations, especially in agricultural areas [22,23,24]. Sneller et al. [25] reported that approximately 85 tons of neodymium (Nd) were released into the environment from phosphate fertilizer production in the Netherlands in 1994. Slooff et al. [26] reported that industrial emissions in air and water due to fertilizer production in the Netherlands can contain over 500 mg/kg of REEs. Petroleum refining processes can release similar amounts of REEs into the environment [26]. Studies have indicated that REEs can be absorbed by plants due to the similar ionic radii that they share with calcium [27,28]. As a result, REEs may replace calcium molecules in a number of physiological processes involving proteins and enzymes, including root growth, photosynthesis and flowering [28,29]. One of the potential ways to reduce the harmful impact of the excessive amount of REE present on plants, and thus on the rest of the living world, is their immobilization into a geopolymer structure. There are reports on the preparation of geopolymers with rare earth tailing, which is a specific tailing containing high content of heavy metals, and they propose a new scheme of disposing of the rare earth tailing through fixation during geopolymerization reaction, which could reduce the risk of secondary pollution caused by long-term leaching of heavy metals on condition that direct storage in tailings ponds [21]. 

One of the challenges is the fact that, despite a large number of studies and increasing attention about geopolymers, they are not fully elucidated, and the process of geopolymerization and the structure itself are still not well understood, given the diversity of precursors that can serve as a basis for obtaining these types of materials.

The focus of the present work is to pay more attention to the synthesis as well as the structural and chemical properties of the obtained geopolymer samples. 

Ivanovic et al. examined the thermodynamic parameters (viscosity, density, refractive index, velocity of sound) of the alkaline activator and examined the influence on the synthesis of geopolymers [30]. It was confirmed that, with the knowledge of thermodynamic parameters, there is a better understanding of the first phase of gel formation of geopolymer structure. In previous research by Nenadović et al. [31], Kljajević et al. [32] and Ivanović et al. [33], the synthesis and structure of geopolymer material, the influence of aluminosilicate matrix and alkali activator change were monitored. Materials with 12 M NaOH are the most common subjects, so the aim of this study is to examine the effect of Nd and Sm in the form of oxide incorporation in the first phase of gelation of the geopolymer structure. In addition to the thermodynamic parameters of the alkaline activator 12 M NaOH, the highest influence on the synthesis of geopolymer materials was examined, which is the reason for choosing the incorporation of Nd and Sm into the geopolymer material synthesized with 12 M NaOH.

## 2. Results and Discussion

### 2.1. Thermodynamic Parameters of Alkaline Activator

**Figure 1:**
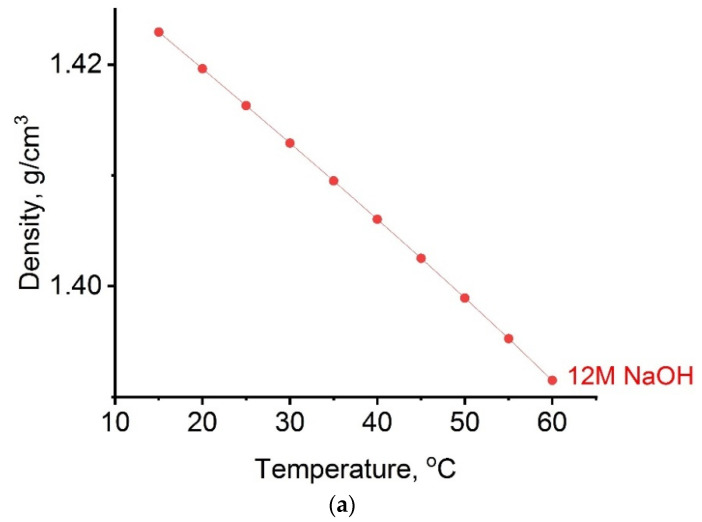

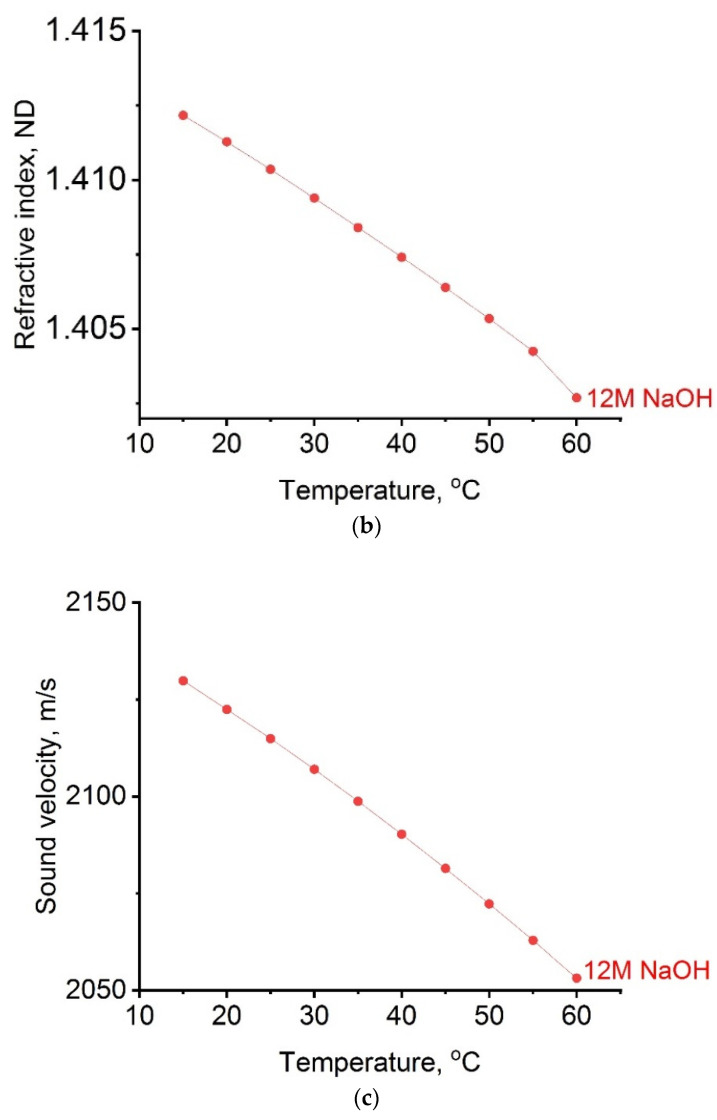
Influence of temperature on: (**a**) density, (**b**) refractive index, (**c**) velocity of sound on alkaline activator.

Densities, refractive index and sound velocity of alkaline activator were determined at the temperature range from 15 to 60 °C. This temperature range covers the process of geopolymerization. Based on the results shown in Figure 1a–c, one can see that, for all tested systems, changes with temperature followed the usual behavior observed in liquid systems; that is, they decrease with increasing temperature due to the liquid’s thermal expansion. Such observation may be attributed to the diminishing intermolecular forces in the liquid brought about by the thermal expansion of the liquid and the increasing velocities of the molecules (or ions) at higher temperatures.

In previous work by Ivanovic et al. [30], velocity of sound values shows a small increase with increasing temperature for solutions with lower NaOH concentration, which may be an indicator of increased ion mobility in solution. Since the changes during heating of the alkaline activator are in the range of 15–60 °C below 2.0% when it comes to density and refractive index, and since the velocity of sounds is slightly above 3.0%, this indicates the thermodynamic stability of the activator leading to dissolution of the initial aluminosilicate types and creation of a new Na–aluminosilicate gel structure of geopolymer samples.

### 2.2. Structural Analysis

**Figure 2:**
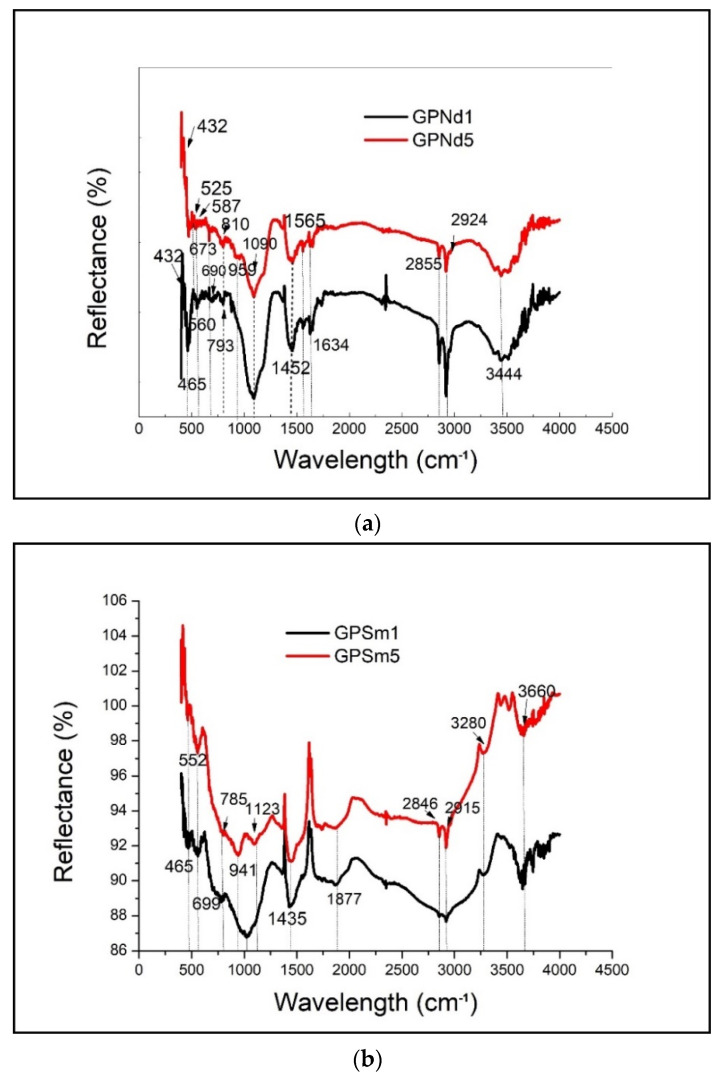
DRIFT spectra of: (**a**) GPNd and (**b**) GPSm.

DRIFT data provided information regarding the energy of bond vibrations that occurred within the sample, and as such could distinguish between Si-O-Si and Si-O-Al bonds and allow observation of the location of nonbridging oxygen sites [34] and the presence of carbonate species, which may not be readily apparent in the solid-state XPS data. Figure 1 shows the DRIFT spectra for geopolymer samples doped with 1.0 and 5.0 wt.% of two rare earth oxides Sm_2_O_3_ and Nd_2_O_3_, denoted as GPE1 and GPE5 (E is Nd or Sm), respectively. The DRIFT spectrum for GPNd1 and GPNd5 (Figure 2a) exhibits a high intensity band at 1090 cm^−1^ that is assigned to asymmetric stretching of Si-O-T bonds [28,32,35,36,37,38,39,40,41] (where T = Al or Si in tetrahedral coordination) and a low-intensity band at 810 cm^−1^ that is assigned to symmetric stretching of Si-O-T bonds [41]. The bands at ~460 cm^−1^ can be related to the Si-O-Si bending mode, and reflectance at 560 cm^−1^ can be attributed to the Si-O-Al vibrations. The bands at 690 cm^−1^ can be attributed to the Si-O-Si symmetric stretching vibrational mode. Si-O banding vibrations are responsible for the bands occurring at 793cm^−1^ (corresponds to quartz). The reflectance at 923 cm^−1^ corresponds to Si-O bending vibrations [41]. It has been known that the peaks located at about 3444 cm^−1^ and 1634 cm^−1^ correspond to the stretching and contracting vibrations of -OH in water [42,43,44]. The tense peaks of 1090 cm^−1^, 810 cm^−1^ and 465 cm^−1^ in geopolymers are caused by vibration of Si-O-Si, Al-O and O-Si-O, respectively [45], revealing that the geopolymer contains a large amount of active Si and Al components. The peaks 1452 cm^−1^ in geopolymers were related to the symmetrical vibration of the O-C-O in CO_3_^2−^ [46,47]. 

The peaks at 432 cm^−1^ and 525 cm^−1^ correspond to the characteristic Nd-O vibrations of Nd_2_O_3_ nanoparticles [48]. The incorporated Nd samples exhibit the presence of bands at 587 cm^−1^ and 673 cm^−1^ and also correspond to Nd-O vibrations of Nd oxides [49]. The spectrum has an enormous number of weak absorption peaks, which indicates weak O-H vibrations and sharp peaks for strong O-H vibrations. Moreover, reflectance at 1565 cm^−1^ is new in the geopolymer system and is possible to connect with the Nd_2_O_3_ structure. 

The existence of bands at 525 cm^−1^ and 685 cm^−1^ was observed in geopolymer samples with 5% of Sm_2_O_3_ (Figure 2b); these two bands could be attributed to the stretching vibration of Sm_2_O_3_ species and bending vibration of Sm-O-H groups, respectively [50]. A noticeable band at 785 cm^−1^ due to the stretching vibration of Sm^3+^ -O groups in Sm_2_O_3_ phase is observed in the case of GPSm5. An intense wide band is observed around 1028 cm^−1^ due to Sm^3+^ (stretching vibration) [51] ion doping in the prepared sample. This band is wide and most likely overlaps with the Si-O band, which belongs to the basic geopolymer structure found in this range [31]. The presence of samarium oxide in the geopolymer samples improves the optical properties of sample.

The peaks shown in Figure 2b corresponding to H-O-H, -OH, Si-O-T (T-Si, Al), Si-O, O-C-O, and the presence of the organic phase of geopolymer structure of the samples GPSm1 and GPSm5 are 3280, 3660, 465, 552, 699, 1028, 1123, 1435, 2846, 2915 cm^−1^.

### 2.3. XRD Analysis 

**Figure 3:**
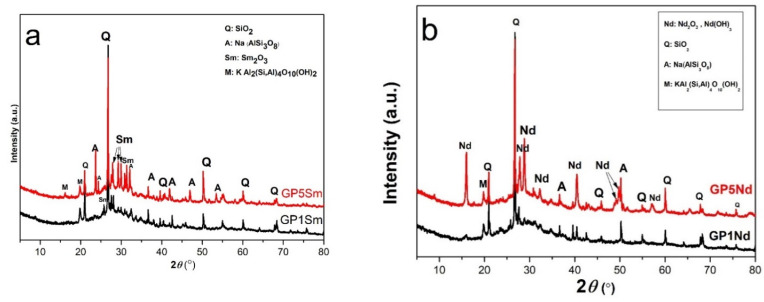
XRPD diffractograms of samples: (**a**) GP1Sm and GP5Sm and (**b**) GP 1Nd and GP5Nd.

As can be seen from the results of X-ray diffraction in both samples presented in Figure 3a,b, the existence of crystalline albite quartz and some muscovite peaks is evident, indicating semicrystalline structural formation. During geopolymerization process and synthesis reaction, aluminosilicate mineral phases stay unchanged. Sample GP1Sm is characterized by significantly lower intensities of Sm peaks in contrast to sample GP5Sm, where peaks are significantly more intense and sharper. The increase in the intensity and sharpness of the peaks in GP5Sm indicate that the contribution of Sm and its incorporation into the geopolymer matrix is higher, which is in correlation with the synthesis procedure.

It is also evident, by comparing the diffractograms, that the peaks are slightly shifted to the left, which indicates the incorporation of Sm into the primary geopolymer gel. Increasing of intensities of Sm peaks on presented diffractograms is related to higher mass volume addition of Sm during the synthesis, which is unequivocally confirmed by obtained XRD results. Based on XRD results presented in Figure 3b, it is clear that Nd in geopolymer matrix is represented as joint contribution of oxide and hydroxide in geopolymer gel. By comparing the results of XRD analysis in Figure 3b, it is clear that the GP5N sample with a higher percentage mass volume of neodymium addition in the synthesis process reveals peaks with significantly higher intensity, indicating confirmation that a larger amount of Nd is incorporated in the geopolymer structure. As in the previous samples, the main crystalline phases of the geopolymer remain unchanged. A high background in the range of 10 to 40° 2θ indicates the formation of a primary geopolymer matrix in all samples.

### 2.4. XPS Analysis

**Figure 4:**
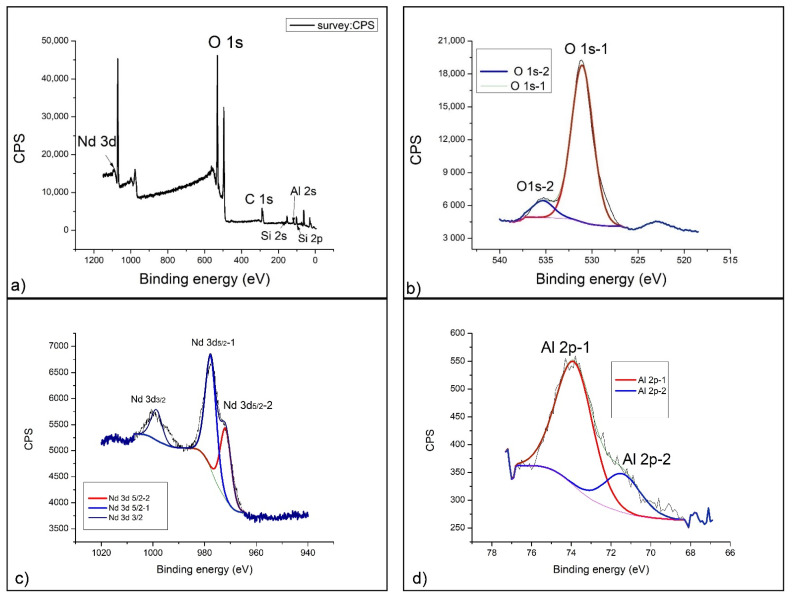

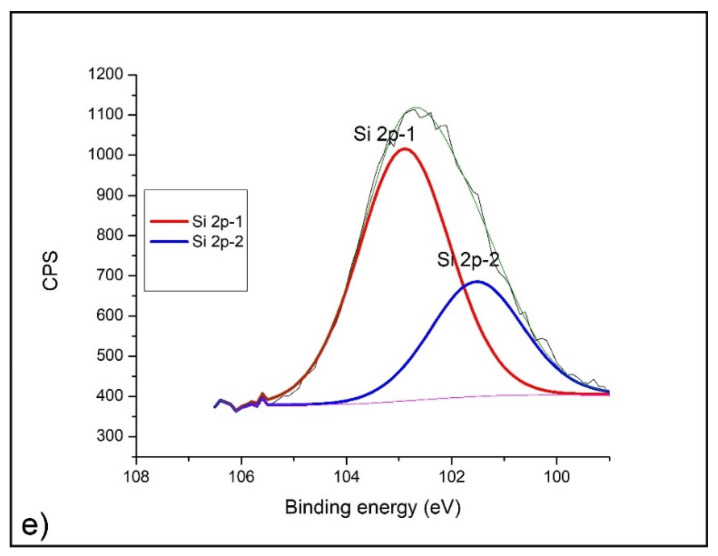
XPS spectrum of GP with 1% Nd: (**a**) Survey spectrum, (**b**) O 1s, (**c**) Nd 3d, (**d**) Al 2p and (**e**) Si 2p.

Figure 4a presents a survey spectrum of a geopolymer doped with 1% neodymium. The most dominant peaks observed are O1s and C1s, on the basis of which further detailed analysis of the spectrum was performed. Since it is an aluminosilicate material, the presence of Al 2p and Si 2p spectral lines is observed in the low-energy part of te spectrum. In the high-energy part of the spectrum (about 1000 eV), a complex peak of the neodymium Nd 3d spectral line appears.

Figure 4b gives a clearer insight into the way of oxygen binding by the detailed spectrum, on which the deconvolution of the O 1s spectral line was performed. The deconvolution of the oxygen spectral line results in two contributions: The first, more dominant (O 1s-1), is located at 531.2 eV of the binding energy and basically represents the dominant phase of Al_2_O_3_. The second contribution, of lower intensity (O 1s-2), is at 535.4 eV of binding energy, and since it is at the end of the energy range of the oxygen spectral line, it belongs to complex aluminosilicate compounds, which, after alkaline activation, are formed as side products (CaAl_2_O_4_ and SiAl_2_O_4_). 

Neodymium (III) oxide, which was used for doping geopolymers, was incorporated into the geopolymer structure forming two dominant contributions of the Nd 3d spectral line (Figure 4c). The first and more dominant contribution is located at a binding energy of 998.9 eV (Nd 3d 5/2-1) and corresponds to the existence of an equilibrium mixture of Nd(OH)_3_ + Nd_2_O_3_, which was formed by alkaline activation. Since hydroxides of rare earths (including neodymium) are not stable, another contribution of the spectral line on 977.9 eV (Nd 3d 5/2-2) occurs [52]. It belongs to pure Nd_2_O_3_, which was the starting material. By quantitative analysis of the areas under the curves, we can conclude that the amount of reacted Nd_2_O_3_ is about two-thirds of the total amount added. One-third of the Nd_2_O_3_ did not react with the alkaline activator at all and remained in the form of the starting oxide. 

The detailed spectrum of the aluminum 2p line further confirms the existence of Al_2_O_3_ at a binding energy of 73.9 eV–Al 2p-1 (Figure 4d). This more dominant part of amorphous Al_2_O_3_ is complemented by the less present crystalline Al_2_O_3_, which is located at a binding energy of 71.4 eV (Al 2p-2) and represents the alpha phase crystal structure [53]. The total ratio of amorphous and crystalline Al_2_O_3_ is 4:1. This is certainly a consequence of the process of alkaline activation, which has a nonselective effect on the starting substances in the geopolymerization reaction. 

By detailed analysis of the Si 2p spectrum, we can confirm the existence of complex structures within the geopolymer. In Figure 4e, one can observe that, at 102.9 eV (Si 2p-1), the SiO_2_ and Al_2_OSiO_4_ phases dominate, with a smaller contribution on 101.5 eV (Si 2p-2) corresponding to the molecular sieve (zeolite 3A) of NaAl_2_SiO_4_ [54]. The chemical composition of the geopolymer formed in this case corresponds to the position of the spectral line for zeolite 3A, only in this case, a predominantly amorphous structure is obtained.

**Figure 5:**
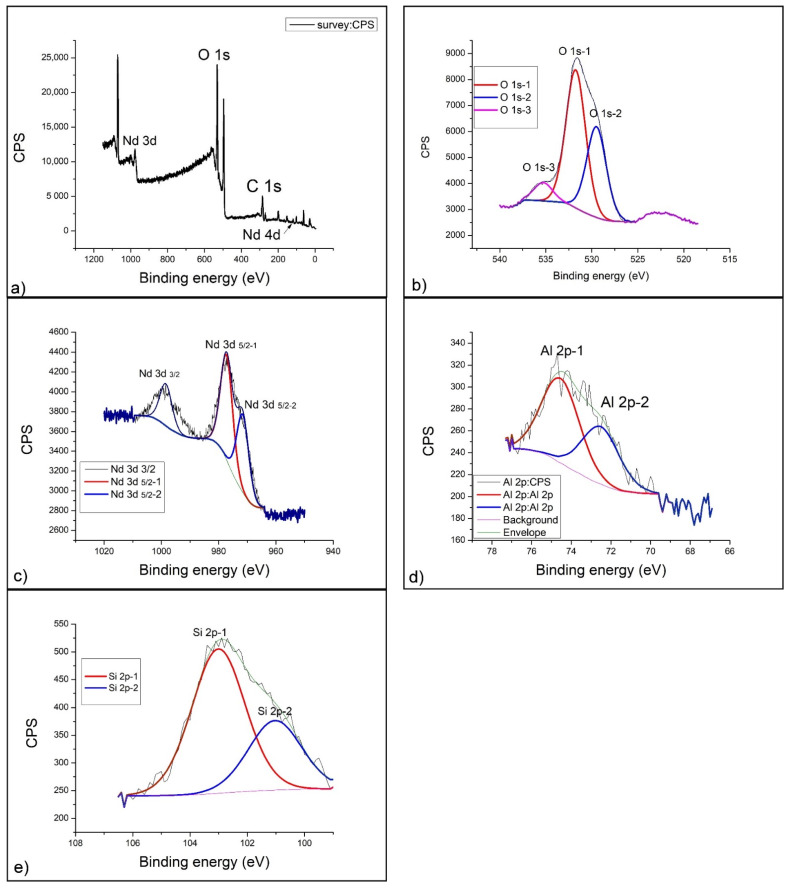
XPS spectrum of GP with 5% Nd: (**a**) Survey spectrum, (**b**) O 1s, (**c**) Nd 3d, (**d**) Al 2p and (**e**) Si 2p.

In the case of the geopolymer doped with 5% neodymium, the appearance of the Nd 4d spectral line together with the dominant Nd 3d is observed on the survey spectrum, which is analyzed in detail (Figure 5a). The appearance of the Nd 4d line in the low-energy part of the spectrum is definitely a consequence of the dopant concentration, i.e., amounts of neodymium present in the geopolymer. The positions of O 1s and C 1s on the survey spectrum are the same as in GP with 1% Nd.

Unlike GP 1% Nd, this sample shows a more complex spectral line O 1s, where deconvolution yields three contributions. The most dominant peak occurring at 531.8 eV O 1s-1 is amorphous Al_2_O_3_. The second most common is the contribution of O 1s-2 to 529.5 eV, and it is related to crystalline Al_2_O_3_. In Figure 5b, it can be determined that the ratio of amorphous to crystalline Al_2_O_3_ is 2:1. The difference in relation to GP 1% Nd is the appearance of the crystalline phase Al_2_O_3_ in addition to the dominant amorphous one. At a binding energy of 535.4 eV (O 1s-3), as in the previous case, a clear presence of CaAl_2_O_4_ and SiAl_2_O_4_ as a side product of geopolymerization is confirmed. 

Detailed analysis of the Nd 3d spectral line in the case of GP 5% Nd does not reveal any new behavior. The more dominant contribution of Nd 3d 5/2-1 to 998.9 eV related to Nd(OH)_3_ is in equilibrium with the contribution to 977.9 eV (Nd 3d 5/2-2) and refers to pure Nd_2_O_3_ (Figure 5c). The ratio of the amounts of hydroxide and oxide phase remains unchanged. 

A detailed analysis of the Al 2p spectral line shows a slight shift in the contribution to higher values of the binding energy (Figure 5d). The more dominant contribution of Al 2p-1 at 74.6 eV and the weaker contribution at 72.5 eV refer, as in the previous case, to the amorphous and alpha crystalline phase [54]. The only thing that has changed is the ratio in the composition of these two phases, and it is 2:1. In the geopolymer sample with 5% Nd, the amount of the amorphous phase Al_2_O_3_ decreased, while the quantity of alpha crystalline phase remained almost unchanged (Figure 5e).

**Figure 6:**
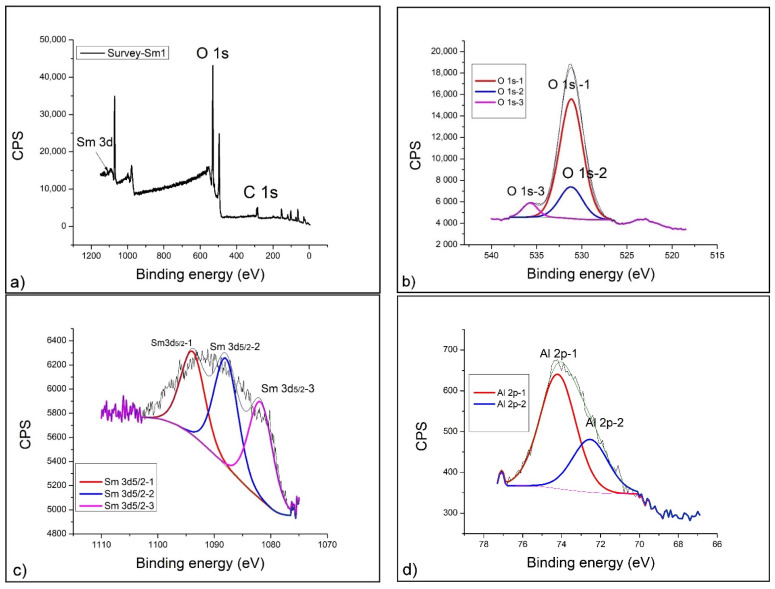

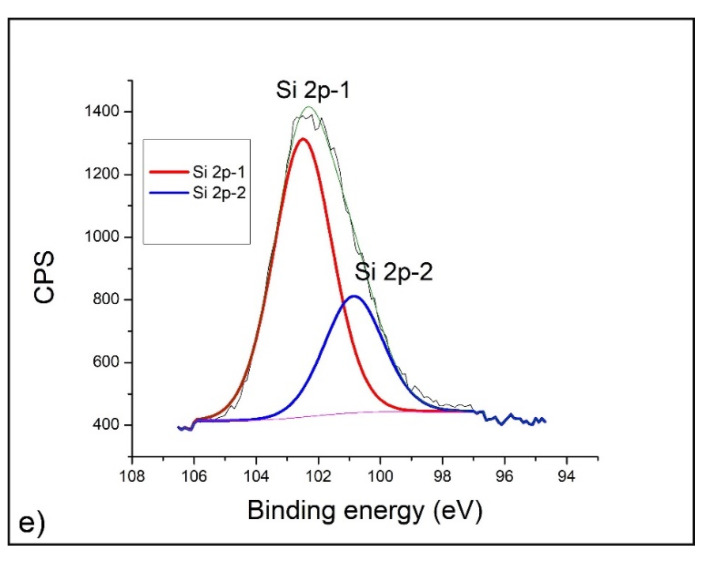
XPS spectrum of GP with 1% Sm: (**a**) Survey spectrum, (**b**) O 1s, (**c**) Sm 3d, (**d**) Al 2p and (**e**) Si 2p.

Figure 6a presents a survey spectrum of geopolymers with 1% added samarium. At first glance, it can be seen that the amount of carbon has been reduced, but the positioning of the energy axis has been performed without any problems. In the high-energy part of the spectrum over 1000 eV, the spectral line of Sm 3d is observed.

The position of the oxygen spectral line O1s -1 is at the expected place. Detailed analysis (Figure 6b) of this spectral line shows a dominant peak at 531.2 eV corresponding to Al_2_O_3_. Similar to the other samples, the weaker peak at 535.7 eV corresponds to CaAl_2_O_4_ and SiAl_2_O_4_. The ratio of amorphous to crystalline Al_2_O_3_ also remains unchanged and is 2:1. 

Figure 6c represents the detailed spectrum of the Sm 3d line. For the purposes of the analysis, only a 3d_5/2_ spectral line was used, because it gives a complete insight into the behavior of Sm_2_O_3_ when it is affected by a strong base in the process of alkaline activation. Similar to GP with neodymium, samarium also shows a similar tendency to form an unstable hydroxide at a binding energy of 1088.1 eV (Sm_2_O_3_ + Sm(OH)_3_ mixture-Sm 3d_5/2_-2) as well as the initial constituent Sm_2_O_3_ at 1081.9 eV-Sm 3d_5/2_-1 [55].

Detailed spectra of Al 2p and Si 2p spectral lines (Figure 6d,e) indicate the formation of a complex geopolymer matrix of aluminosilicates. Thus, at a binding energy of 74.2 eV (Figure 6d), the formation of a complex crystal hydrate of Al_2_Si_2_O_7_. 2H_2_O can be noticed with the existence of an amorphous phase of Al_2_O_3_, which is almost always present [56].

The aluminosilicate base of the geopolymer served as a good matrix for incorporation of rare earths (Nd and Sm), and XPS analysis showed that the behavior of these elements is similar in terms of alkaline activation but that neodymium is somewhat more sensitive to strong bases and more transformed into unstable hydroxide Nd(OH)_3_. Samarium (III) oxide (Sm_2_O_3_) is therefore slightly less soluble in 12M NaOH and, to a smaller extent, converted to hydroxide Sm(OH)_3_. The XPS spectral analysis provided full-insight chemical structure information of the Nd- and Sm-doped GP, as shown in Figure 4, Figure 5 and Figure 6. The intensity of peaks corresponding to Si, O and Al elements changed, indicating the transformation of chemical bond during the doping process. The intensification of O, Si, and Al peaks occurs mainly due to the geopolymerization of aluminosilicate. There are three types of primary chemical states of oxygen in geopolymer including Si-O-Al, Si-O-Si and Si-O-H, with the main spectral line positions at 531, 532 and 533 eV, according to the literature [57,58,59]. The fitted parameters of the O1 s XPS spectra are shown in Figure 4, Figure 5 and Figure 6b for GPNd and GPSm, respectively. There are more Si-O-Al and Si-O-H chemical bonds in GPSm, which is in accordance with the FTIR results. 

### 2.5. TEM Analysis

**Figure 7:**
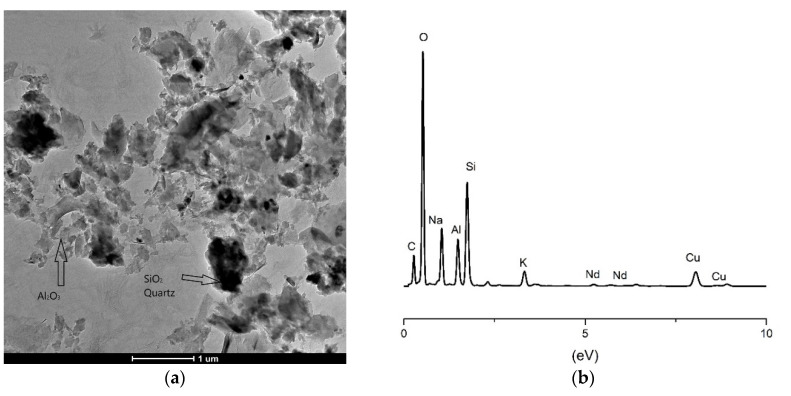
TEM analysis of geopolymer: (**a**) with 5% Nd; (**b**) corresponding EDS analysis.

**Figure 8:**
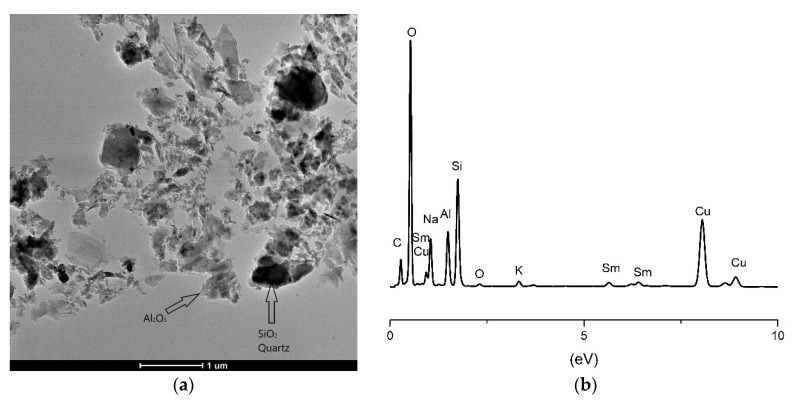
TEM analysis of geopolymer: (**a**) with 5% Sm; (**b**) corresponding EDS analysis.

The geopolymer samples incorporated with 5% of Nd_2_O_3_ and Sm_2_O_3_ were additionally analyzed using transmission electron microscopy (TEM). The given images at magnification 11kx of both samples and X-ray energy dispersive spectrum (EDS) that is acquired on the same images are shown in Figure 7 and Figure 8.

In Figure 7a, one can see the existence of two completely different phases, which are contrastingly divided as light and dark fields. Lighter-colored grains are primarily related to Al_2_O_3_. The dark grains in the image represent SiO_2_ in the form of quartz. Other zones, which are in different shades of gray, are a mixture of the most common phases as well as other aluminosilicate phases. Individual grains are in the range of 50 to 100 nm, but due to the occurrence of aggregation, they form larger grains of about 500 nm. The presence of Nd as a dopant can be confirmed by EDS analysis (Figure 7b), where alpha and beta lines appear in the middle of the energy axis. By structural analysis, we can achieve a clear insight into the incorporation of Nd as a dopant. It partly entered the structure but partly remained as a separate phase at the grain boundaries.

The situation is similar with geopolymers doped with 5% Sm (Figure 8a). A clear division into light and dark fields indicates the existence of Al_2_O_3_ and SiO_2_ (quartz). The separation of the phase of neodymium and samarium oxides can be explained by insufficient reactivity and the absence of thermodynamic parameters for all Nd^3+^ and Sm^3+^ ions to be incorporated into the geopolymer structure. The partial ion change occurred, but the solid solution was not completely formed. Comparing these results with the XPS results, we can conclude that we have two dominant phases of Nd and Sm that chemically belong to hydroxides Nd(OH)_3_ and Sm(OH)_3_ and oxides as the starting dopant. 

The particle size presented in Figure 8a shows a wide distribution from smaller grains to larger clusters. This behavior is a consequence of the reaction path that leads to the final product. The reaction in a solution in which a strong base is present leads to the typical micro-nano structures that are formed. Smaller particles that are initially formed combine with larger ones in the process of geopolymerization and coalesce into larger crystals that are multiphase. In this way, Nd^3+^ and Sm^3+^ ions, whose ionic radius is much larger than the ionic radius Al and Si, have a high resistance to entering the aluminosilicate lattice [60] and are only partially incorporated, while they are distributed thermodynamically more stable at the grain boundaries. Partially dissolved aluminosilicates in a concentrated alkaline medium form an amorphous geopolymeric gel incorporated with undissolved crystalline particles. Some aluminosilicates dissolve preferentially to form an equilibrium ratio of aluminum to silicon in the gel. As a consequence, the gel phases formed during the geopolymerization of the kaolinite, Nd and Sm are probably amorphous to semicrystalline in structure. This may be due to the varied Si/Al ratio of raw material and different polymerization conditions [61,62]. On both TEM images (Figure 7a and Figure 8a), formed gels have a relatively consistent shape and size of primary particles with neodymium and samarium, respectively. The particle size is slightly larger in GPSm (Figure 8a), about 15–20 nm larger compared to GPNd (Figure 7a). Phair et al. report particles in the order of 10 nm in diameter for all compositions of similar aluminosilicate gels studied, also noting a slight increase in particle size as sodium concentration increases [63]. Chen and Mondal observed gel particle size to increase and appear smoother and rounder with the addition of NaOH [64]. Here the difference of sodium content between gels is not so significant, but particles with higher sodium concentrations are larger and smoother. 

## 3. Conclusions

The incorporation of neodymium and samarium ions into the geopolymer matrix was the aim of this research. A better doping effect was achieved with a concentration of 5 wt.% than with 1 wt.%. This innovative process has shown that there are certain structural and chemical changes in the geopolymer matrix. Trivalent Nd^3+^ and Sm^3+^ ions, which formed a nonequilibrium charge in GP, formed Nd-O and Sm-O bonds confirmed by the DRIFT method, where the structural change in the geopolymer aluminosilicate matrix occurred. The phase composition of the newly obtained composite gel confirmed the existence of two dominant phases of neodymium and samarium in GP. They were mutually confirmed by XRD and XPS methods, where the starting oxide and unstable hydroxide were detected in equilibrium. The rest of the dominant phase is the aluminosilicate matrix of GP with a variable Al-to-Si ratio in the system. Transmission microscopy revealed an even clearer insight into the micronano structure of the resulting gel composite. Smaller crystallites coalesce into larger grains under the influence of a strong base in the geopolymerization process, two different phases of Al_2_O_3_ and SiO_2_ (quartz) are clearly visible, while Nd and Sm precipitates have separated at the grain boundaries.

## 4. Materials and Methods 

### 4.1. Materials

Geopolymer gels were produced by reaction of metakaolin described by Nenadović et al., 2017 [31] with a sodium silicate-activating solution. The activating solutions were prepared by dissolving 12 M sodium hydroxide powder (Sigma–Aldrich, St. Louis, MO, USA, analytical grade) and sodium silicate (technical grade) in appropriate ratio (volume ratio Na_2_SiO_3_/NaOH = 1.5). 

Commercially available Nd_2_O_3_ (Acros Organics, Geel, Belgium, 99.9%) and Sm_2_O_3_ (Merck KGaA, Darmstadt, Germany, 99+) powder was mixed during process of geopolymerization in geopolymer paste. The two weight fractions of both of these oxides (1% and 5%) were used in process of preparation of geopolymer samples. Metakaolin/Nd_2_O_3_, Sm_2_O_3_ and the alkaline solution (solid/liquid ratio was 1.0) were mixed for 15 min and then left at room temperature for one day. After that, the mixture was kept at 60 °C for additional two days in appropriate covered molds and subsequently aged at room temperature in controlled conditions for 28 days. 

### 4.2. Methods and Characterization

- Thermodynamic parameters of activating solution were examined. Using the Anton Paar DSA 5000 M digital densitometer, the density as well as the speed of sound were measured. The range of density measurements on this device is from 0 to 3 × 10^−3^ kgm^−3^, and the speed of sound is from 1000 to 2000 m s^−1^. Experimental measurement of refractive index was performed on an automatic refractometer (model Anton Paar RXA 156), which operates at a wavelength of 589 nm. The device has a built-in thermostat with an accuracy of ±0.03 K by which the temperature of the samples is kept constant during the measurement. The measurement range of the refractive index is from 1.32 to 1.56. For all thermodynamics parameters, all measurements were performed in the temperature range from 288.15 K to 333.15 K.
- Diffuse reflectance infrared Fourier transform spectroscopy (DRIFTS) is a cheap, fast and nondestructive way of evaluating clay minerals and their products [25]. Drift spectra were obtained using the Perkin–Elmer FTIR spectrometer. Approximately 5% samples were dispersed in oven-dried spectroscopic grade KBr with the refractive index of 1.559 and particle size of 5–20 µm.

Background KBr spectra were obtained and spectra were rationed to the background. The spectra were scanned at 4 cm^−1^ resolution and collected in the mid-IR region from 4000 to 400 cm^−1^.

- XRD measurements were conducted at room temperature using Ultima IV Rigaku diffractometer, equipped with CuKα1,2 radiation, using a generator voltage (40.0 kV) and a generator current (40.0 mA). The range of 5–80° 2θ was used for all powders in a continuous scan mode with a scanning step size of 0.02° and at a scan rate of 5°/min using D/TeX Ultrahigh-speed detector. Samples were crushed in the in a porcelain mortar to the fineness of a fine powder. Si–monocrystalline sample carrier was used. The PDXL2 (Ver. 2.8.4.0) software was used to evaluate the phase identification and microstructure properties of material [62,63]. All obtained powders were identified using the ICDD data base [64]. For phase identification, selected PDF card numbers were used: Quartz (SiO_2_; 01-079-6237), Albite (Na(AlSi_3_O_8_); 01-084-0982), Muscovite (KAl_2_(Si,Al)_4_ O_10_(OH)_2_; 00-058-2036), Samarium Oxide (Sm_2_O_3_; 00-042-1464), Neodymium Hydroxide Nd (OH)_3_, 01-070-0214) and Neodymium oxide (Nd_2_O_3_, 00-006-0408).
- XPS analysis was performed using a SPECS instrument for detailed chemical composition characterization using X-ray-induced photoelectron spectroscopy. More detailed explanation can be found in Nenadovic et al., 2017 [31] and Ivanovic et al., 2020 [33]. Photoelectron emission was excited by monochromatic Al Kα line with photon energy of 1486.67 eV. Detailed spectra of the main photoelectron lines were taken in the fixed analyzer transmission mode with a pass energy of 20 eV (FAT 20), an energy step of 0.1 eV and a dwell time of 2 s. Charging compensation was performed using an electron flood gun and the constant current and voltage. The binding energy axis was adjusted according to the position of the carbon C 1s line. The survey spectra were performed according to the characteristic spectral line intensities. Specific atomic sensitivity factors for each analyzed element were used to eliminate the background lines, provided by the manufacturer. The photoelectron lines were fitted to peaks using appropriate software package.
- Characterization and investigation of the micronano samples structure was carried out by TEM, using a FEI Talos F200X microscope operating at 200 keV. A CCD camera with a resolution of 4096 × 4096 pixels was used for acquiring micrographs using the User Interface software package. The geopolymer samples were also further analyzed using scanning transmission (STEM) mode with energy dispersive spectrometry (EDS). The EDS detection system was used to determine the presence of doping species of Nd and Sm in geopolymer matrix. High-angle annular dark-field (HAADF) imaging was used in nanoprobe–TEM mode with a camera length of ~200 nm using the standard annular dark-field detector. The powder samples were prepared by standard rinsing and diluting in ethanol to a sufficient concentration to trap the geopolymer powder on the TEM grid, dried in air and then transferred to microscope.

## Data Availability

We exclude this statement because we don’t have any data.
